# Combined effect of functional impairment and metabolic dysregulation on post-stroke depression risk

**DOI:** 10.3389/fpsyt.2026.1843802

**Published:** 2026-10-16

**Authors:** Tianhao Zhang, WenLu Shi, Qingjie Chi, Jinhua Qian, Tianle Wang, Li Zhu, Wenbing Ding, Zhuo Chen

**Affiliations:** 1 Department of Radiology, Affiliated Nantong Clinical College of Nantong University, Nantong First People’s Hospital, Nantong University, Nantong, China; 2 Department of Neurology, Nantong First People’s Hospital, Southeast University, Nantong, China; 3 Department of Intervention, Affiliated Nantong Clinical College of Nantong University, Nantong First People’s Hospital, Nantong University, Nantong, China

**Keywords:** functional impairment, metabolic dysregulation, post-stroke depression, stroke, triglyceride-glucose index

## Abstract

**Background:**

Post-stroke depression (PSD) is one of the most common neuropsychiatric complications among stroke survivors, significantly affecting rehabilitation adherence and long-term clinical outcomes. Although previous studies have examined the roles of either functional impairment or metabolic dysregulation in PSD, limited research has explored their combined impact from an integrative perspective.

**Methods:**

This study included 245 stroke patients, of whom 65 were diagnosed with PSD. Restricted cubic spline (RCS) analysis was used to assess nonlinear associations between the atherogenic index of plasma (AIP), triglyceride-glucose (TyG) index, and the risk of PSD. Multivariate logistic regression was performed to identify independent predictors. A partial least squares (PLS) model was constructed using four variables—Barthel index, body mass index (BMI), AIP, and TyG index. Then the K-means clustering was applied to identify functional–metabolic subtypes and examine their associations with PSD.

**Results:**

Compared with non-PSD patients, those with PSD showed significantly lower Barthel index scores and higher TyG and AIP levels. RCS analysis indicated a nonlinear relationship between AIP and PSD. Logistic regression identified the Barthel index (OR = 0.97, 95% CI: 0.95-0.99) and TyG index (OR = 2.04, 95% CI: 1.19-3.47) as independent predictors of PSD. PLS-K-means clustering revealed four distinct subtypes. Patients in subtype 3 (metabolically susceptible subtype) and subtype 4 (dual-impaired subtype) had significantly higher PSD risks compared to subtypes 1 and 2 (OR = 2.13 and 2.63, respectively).

**Conclusions:**

Functional impairment and metabolic dysregulation are jointly associated with the development of PSD, and their interaction may constitute a key potential mechanistic.

## 1 Introduction

Stroke remains the second leading cause of mortality and disability worldwide, often resulting in poor long-term outcomes. It not only substantially compromises patients’ quality of life but also imposes significant economic burdens on families and healthcare systems (1). Post-stroke depression (PSD) is among the most common neuropsychiatric complications in stroke survivors. Systematic reviews and epidemiological studies have consistently shown that approximately one-third of stroke survivors experience depressive symptoms (2–4). PSD is clinically characterized by persistent low mood, anhedonia, fatigue, and impaired concentration. In severe cases, it can significantly undermine rehabilitation adherence, impede neurological recovery, and worsen long-term functional outcomes (5, 6). Furthermore, PSD is independently associated with increased risks of stroke recurrence, functional decline, and mortality, making it a key determinant of poor post-stroke prognosis.

As a highly disabling neurological disorder, stroke frequently leads to varying degrees of impairment in activities of daily living (ADL) (7). These functional deficits not only limit the autonomy of stroke survivors but also exacerbate psychological stress and increase dependency on caregivers (8). Accordingly, impaired functional status has been consistently associated with a higher likelihood of PSD. Prior research has shown that greater functional impairment is associated with elevated psychological distress and caregiver reliance, thereby increasing the likelihood of depressive symptoms (9). The Barthel Index is a widely adopted tool for assessing ADL performance in stroke patients. It evaluates basic daily functions such as eating, dressing, toileting, and ambulation, and is routinely used to monitor functional recovery during rehabilitation (10). Studies suggest that patients with Barthel scores above 60 are more likely to regain independence, actively participate in community life, and report higher levels of life satisfaction (11). Moreover, a significant inverse relationship has been identified between Barthel Index and PSD risk, indicating that lower functional independence is strongly associated with increased vulnerability to depression (12).

In addition to functional impairment, accumulating evidence suggests that metabolic dysregulation is associated with a range of clinical outcomes following stroke. Emerging metabolic markers—such as the Atherogenic Index of Plasma (AIP) and the Triglyceride-Glucose (TyG) index—capture individual variability in lipid metabolism and insulin resistance, and are increasingly recognized as valuable tools for risk stratification in cardiovascular and cerebrovascular diseases. Specifically, AIP serves as a novel indicator of dyslipidemia and atherosclerotic risk (13), while the TyG index is a reliable surrogate for insulin resistance and glucose metabolism dysfunction (14). Elevated levels of AIP and TyG are often indicative of metabolic dysregulation, including hyperglycemia and hyperlipidemia, which have been linked to chronic low-grade inflammation, disruption of the blood–brain barrier, and neurotoxic cascades in the post-stroke period (15, 16). These findings provide a novel metabolic lens for understanding the pathophysiology of post-stroke mood disorders.

Current evidence suggests that both functional impairment and metabolic dysregulation are closely associated with post-stroke depression. Nevertheless, how these two domains interact to shape PSD risk remains poorly understood. To bridge this gap, we systematically explored PSD from both functional and metabolic perspectives—integrating Barthel Index–based functional status with AIP- and TyG-derived metabolic burden. Our goal is to clarify their combined impact on PSD, thereby offering a theoretical basis for early risk stratification and individualized intervention in stroke survivors.

## 2 Methods and materials

### 2.1 Participants

This single-center retrospective study included 245 consecutive patients admitted with acute ischemic stroke between June 2023 and December 2024. Inclusion criteria: (1) magnetic-resonance confirmation of acute ischemic cerebral infarction; (2) age 18–80 years; (3) admission within 7 days of symptom onset; and (4) completion of all requisite examinations with complete clinical data available. Exclusion criteria: (1) non-ischemic stroke; (2) severe disturbances of consciousness, cognition or speech precluding evaluation; (3) pre-existing depression or other psychiatric disorders, or current use of antidepressant/antipsychotic medication; (4) malignancy or other serious comorbid illness; and (5) neurodegenerative disease such as Parkinson’s disease or dementia. The study protocol was reviewed and approved by the Ethics Committee of the Nantong First People’s Hospital (No. 2024KT487). Owing to the retrospective design of the study, the requirement for informed consent was waived by the Ethics Committee.

Demographic and clinical variables—sex, age, height, weight, prior stroke, smoking and alcohol status, hypertension, diabetes, and laboratory indices—were extracted from the hospital information system at admission. Laboratory parameters included neutrophil-to-lymphocyte ratio (NLR), fasting blood glucose (FBG), glycated hemoglobin (HbA1c), total cholesterol (TC), triglycerides (TG), high-density lipoprotein (HDL) and low-density lipoprotein (LDL). Stroke severity was evaluated on admission using the National Institutes of Health Stroke Scale (NIHSS) (4).

### 2.2 PSD

The first month after stroke was predefined as the assessment window for PSD. All participants underwent a single assessment for depressive symptoms within this period after their clinical condition had become relatively stable. The exact timing of assessment was not fixed and varied according to each patient’s clinical stability and ability to complete the evaluation. A neurologist with over five years of clinical experience conducted evaluations using the 24-item Hamilton Depression Rating Scale (HAMD-24). Patients who met the diagnostic criteria for depression based on the Diagnostic and Statistical Manual of Mental Disorders, Fifth Edition (DSM-5) were classified as having PSD (17).

### 2.3 Assessment of functional status

Functional status was assessed using the Barthel index, a widely adopted tool for evaluating activities of daily living (ADL) in stroke patients. This scale includes 10 basic self-care items—such as eating, dressing, toileting, transferring, and walking—and measures the degree of physical independence (18). Total scores range from 0 to 100, with lower scores indicating more severe functional impairment and greater dependence, while a score of 100 reflects full independence.

### 2.4 Assessment of metabolic status

In this study, the AIP, TyG index, and Body Mass Index (BMI) were selected as key metabolic indicators to characterize metabolic dysregulation. AIP is a widely recognized marker of lipid metabolism and is calculated as the base-10 logarithm of the ratio of TG to HDL (19). The TyG index, indicative of insulin resistance, is computed as the natural logarithm of the product of TG and FBG divided by 2 (20). BMI, a commonly used anthropometric measure, reflects the relationship between body weight and height and provides an estimate of overall nutritional status and obesity. It is calculated by dividing weight by the square of height (21). The specific formulas are as follow: $AIP=\;log\left(\frac{TG}{HDL-C}\right)$, $TyG=\ln\frac{TG\times FBG}{2}$, $BMI=\frac{weight}{height^{2}}$. Here, TG, HDL, and FBG are expressed in mmol/L, and weight and height are measured in kilograms and meters, respectively.

### 2.5 Statistical analysis

Statistical analyses were performed using R version 4.4 and SPSS version 27.0. Continuous variables with a normal distribution were summarized as mean ± standard deviation, whereas those with a non-normal distribution were presented as median and interquartile range. Categorical variables were reported as frequencies or percentages. Group comparisons for categorical variables were conducted using the chi-square test, and comparisons for continuous variables were performed using either the independent-samples t-test or the Mann–Whitney U test, depending on distribution characteristics. To investigate potential nonlinear associations between AIP, TyG index, and PSD, RCS analysis was employed. The P value for overall association assessed the overall exposure–PSD relationship, whereas the P value for nonlinearity tested deviation from linearity. Age, sex, NIHSS score, Barthel index, and history of stroke were included as covariates for model adjustment. Knots were placed at the 5th, 35th, 65th, and 95th percentiles of each exposure variable to ensure model flexibility and robustness. The likelihood ratio test was used to assess the presence of nonlinearity. Subsequently, the same set of covariates was included in a multivariate logistic regression model to identify independent predictors of PSD. In addition, PLS regression was conducted using four functional and metabolic variables to extract the first two principal components, capturing potential latent patterns. K-means clustering was then applied in the PLS component space to classify patients into distinct function - metabolic subtypes. These subtypes were further incorporated into a multivariate logistic regression model to evaluate their independent association with PSD risk. A two-tailed P-value< 0.05 was considered statistically significant.

## 3 Results

### 3.1 General characteristics of participants

The baseline characteristics of the study population are summarized in **Table 1**. Among the 245 enrolled stroke patients, 65 (26.53%) were diagnosed with PSD. Compared with non-PSD patients, those with PSD had a significantly higher prevalence of prior stroke history, elevated (NIHSS scores, and lower Barthel index. Additionally, they exhibited significantly higher levels of FBG, TG, AIP, and TyG index (all P< 0.05).

**Table 1:** Baseline characteristics between PSD and non-PSD.

Variables	Non-PSD (n = 180)	PSD (n = 65)	Statistic	*P*
Age, M (Q_1_, Q_3_)	65.50 (57.00, 73.00)	69.00 (58.00, 73.00)	Z=-0.57	0.571
Male, n(%)	126 (70.00)	47 (72.31)	χ^2^ = 0.12	0.726
BMI, Mean ± SD	24.83 ± 3.19	24.43 ± 3.24	t=0.86	0.388
Smoking history, n(%)	65 (36.11)	17 (26.15)	χ^2^ = 2.13	0.145
Alcohol history, n(%)	51 (28.33)	12 (18.46)	χ^2^ = 2.44	0.119
Stroke history, n(%)	40 (22.22)	25 (38.46)	χ^2^ = 6.46	**0.011**
Hypertension, n(%)	152 (84.44)	56 (86.15)	χ^2^ = 0.11	0.741
Diabetes, n(%)	55 (30.56)	26 (40.00)	χ^2^ = 1.92	0.165
NIHSS, M (Q_1_, Q_3_)	2.00 (0.75, 4.00)	3.00 (1.00, 6.00)	Z=-2.34	**0.019**
Barthel index, M (Q_1_, Q_3_)	85.00 (75.00, 90.00)	75.00 (60.00, 90.00)	Z=-3.83	**<.001**
NLR, M (Q_1_, Q_3_)	2.42 (1.83, 3.69)	2.77 (1.96, 3.81)	Z=-0.90	0.371
FBG (mmol/L), M (Q_1_, Q_3_)	5.57 (4.79, 7.69)	6.14 (5.21, 8.90)	Z=-2.47	**0.013**
HbA1c(%), M (Q_1_, Q_3_)	5.80 (5.20, 6.53)	6.10 (5.60, 7.90)	Z=-2.73	**0.006**
TG, M (Q_1_, Q_3_)	1.60 (1.02, 3.48)	1.52 (1.05, 3.52)	Z=-0.38	0.700
TC, M (Q_1_, Q_3_)	3.71 (2.43, 4.35)	3.91 (3.06, 4.49)	Z=-0.90	0.370
HDL, M (Q_1_, Q_3_)	1.23 (1.01, 1.62)	1.18 (0.97, 1.58)	Z=-0.43	0.667
LDL, M (Q_1_, Q_3_)	2.75 (2.13, 4.08)	2.76 (2.11, 4.42)	Z=-0.33	0.738
TyG, M (Q_1_, Q_3_)	3.36 (3.12, 3.75)	3.55 (3.37, 3.98)	Z=-3.35	**<.001**
AIP, M (Q_1_, Q_3_)	0.17 (0.09, 0.27)	0.24 (0.12, 0.40)	Z=-2.47	**0.013**

Values in bold indicate p<0.05. t, t-test; Z, Mann-Whitney test; χ^2^, Chi-square test; SD, standard deviation; M, Median; Q_1_, 1st Quartile; Q_3_, 3st Quartile; BMI, body mass index; NIHSS, national Institutes of Health Stroke Scale; NLR, neutrophil-to-lymphocyte ratio; FBG, fasting blood glucose; HbA1c, glycated hemoglobin; TG, triglycerides; TC, total cholesterol; HDL, high-density lipoprotein; LDL, low-density lipoprotein; TyG, triglyceride-glucose; AIP, atherogenic index of plasma.

### 3.2 Restricted cubic splines

RCS analysis was performed to examine potential nonlinear associations between AIP and TyG index with the risk of PSD (**Figure 1**). The analysis revealed a statistically significant nonlinear relationship between AIP and PSD risk, whereas the association between the TyG index and PSD risk did not reach statistical significance for nonlinearity. Notably, the odds ratio increased more markedly at higher AIP values, with an approximate turning point identified at an AIP value of approximately 0.26, beyond which the risk showed an increasing trend. In the sensitivity analysis excluding the Barthel index from the covariates, the overall association between AIP and PSD remained significant (P for overall = 0.008), although the evidence for nonlinearity was attenuated (P for nonlinear = 0.051); the RCS findings for TyG remained essentially unchanged.

**Figure 1:**
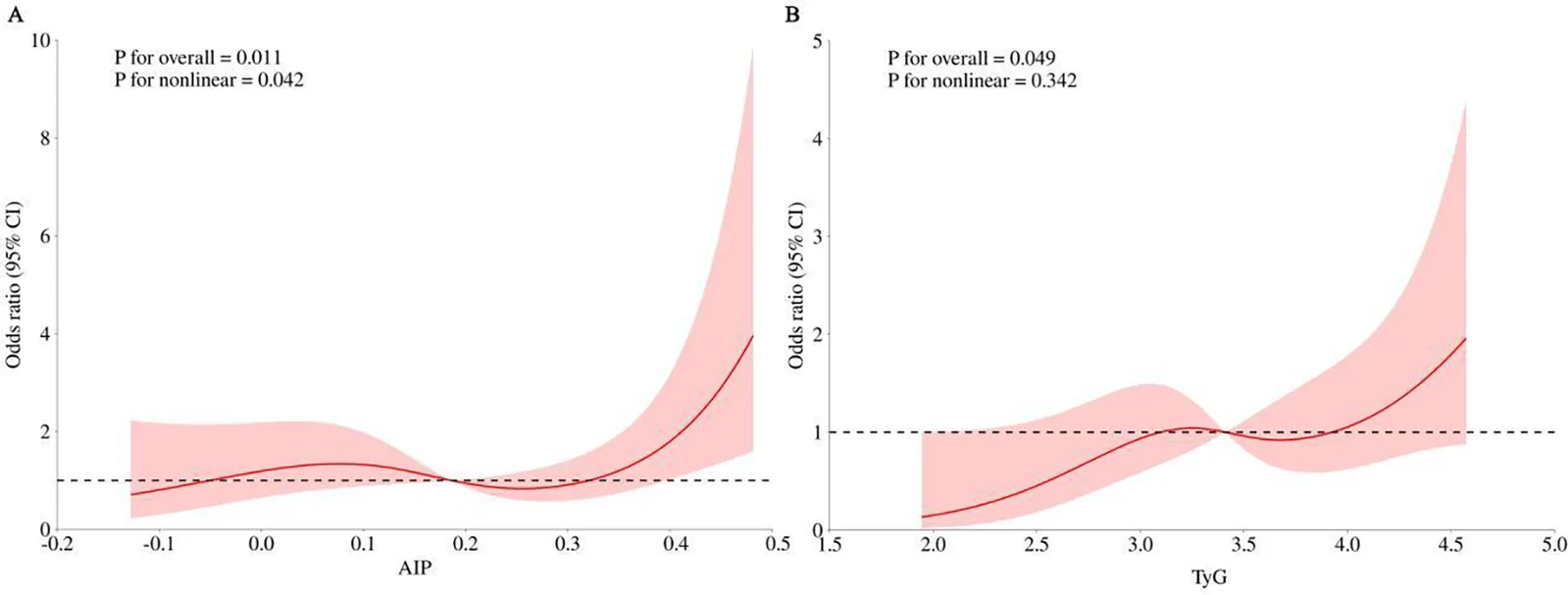
Nonlinear associations between AIP, TyG index and the risk of PSD based on restricted cubic spline analysis. **(A)** AIP showed a statistically significant nonlinear association with PSD risk (P for nonlinearity = 0.042). **(B)** TyG index exhibited a linear association with PSD risk, with no significant evidence of nonlinearity (P for nonlinearity = 0.342). AIP, atherogenic index of plasma; TyG, triglyceride-glucose; PSD, post stroke depression.

### 3.3 Multivariate logistic regression analysis

In the prior RCS analysis, the AIP index exhibited a nonlinear association with PSD risk. To account for this nonlinearity and reduce potential confounding, AIP was categorized into quartiles for the logistic regression analysis. After adjusting for age, sex, and other confounding factors, the Barthel index (OR = 0.97, 95% CI: 0.95–0.99, *P* = 0.039) and the TyG index (OR = 2.04, 95% CI: 1.20–3.47,*P* = 0.009) were identified as independent predictors of PSD (**Table 2**).

**Table 2:** Multivariate logistic regression of PSD.

Variables	β	S.E	Z	*P*	OR [95%CI]
Age	0.00	0.01	0.23	0.821	1.00 [0.98 ~ 1.03]
Male	0.08	0.35	0.22	0.827	1.08 [0.54 ~ 2.15]
NIHSS	0.02	0.06	0.30	0.764	1.02 [0.90 ~ 1.15]
Stroke history	0.54	0.34	1.59	0.113	1.72 [0.88 ~ 3.36]
Barthel index	-0.03	0.01	-2.07	**0.039**	0.97 [0.95 ~ 0.99]
TyG	0.71	0.27	2.61	**0.009**	2.04 [1.19 ~ 3.47]
AIP quantile					
2	-0.63	0.48	-1.31	0.192	0.53 [0.21 ~ 1.37]
3	-0.55	0.48	-1.14	0.254	0.58 [0.23 ~ 1.48]
4	-0.15	0.50	-0.30	0.762	0.86 [0.33 ~ 2.28]

Values in bold indicate p<0.05. OR, Odds Ratio; CI, Confidence Interval; NIHSS, national Institutes of Health Stroke Scale; TyG, triglyceride-glucose; AIP, atherogenic index of plasma.

### 3.4 Identification of subtypes

To integrate functional status and metabolic phenotypes in stroke patients, this study selected the Barthel Index, BMI, AIP, and TyG index as key variables and applied PLS regression to reduce the dimensionality of potential structural features related to PSD. The analysis identified two dominant components (**Supplementary Table S1**): Component 1 was primarily driven by metabolic indicators TyG (loading = 0.657) and AIP (loading = 0.552), reflecting metabolic characteristics; whereas Component 2 was mainly influenced by the Barthel Index (loading =-0.791), representing functional capacity. Together, Component 1 and Component 2 explained 63.13% of the variance in the X-block and 11.84% of the variance in the Y-block, indicating that the retained components captured a substantial proportion of the variation in the predictor space.

Using the two-dimensional functional–metabolic space defined by the PLS components, K-means clustering was performed to identify potential subtypes. The D-index and its second-order differences were calculated across a range of candidate cluster numbers (**Supplementary Figure S1**). The maximum second-order difference was observed at K = 4, supporting the identification of four distinct functional–metabolic subtypes (**Figure 2**):a physiological protection subtype1 (preserved function with optimal metabolic indices), a subclinical burden subtype2 (largely intact function with mild metabolic abnormalities), a metabolic susceptibility subtype3 (acceptable function but markedly elevated TyG and AIP, indicating high metabolic load), and a dual-impaired subtype4 (pronounced functional impairment accompanied by metabolic abnormalities, signifying overall vulnerability). These subtypes differed significantly in functional measures, metabolic status and PSD incidence (**Table 3**). In multivariate logistic regression (**Table 4**), subtype 1 and 2 (good function, low metabolic burden) served as the reference group. Relative to this group, patients in the metabolic susceptibility type (subtype 3) and the dual-impaired type (subtype 4) showed a markedly higher risk of PSD: OR = 2.13 (95% CI 1.02–4.46; p = 0.044) and OR = 2.63 (95% CI 1.16–5.96; p = 0.020), respectively.

**Figure 2:**
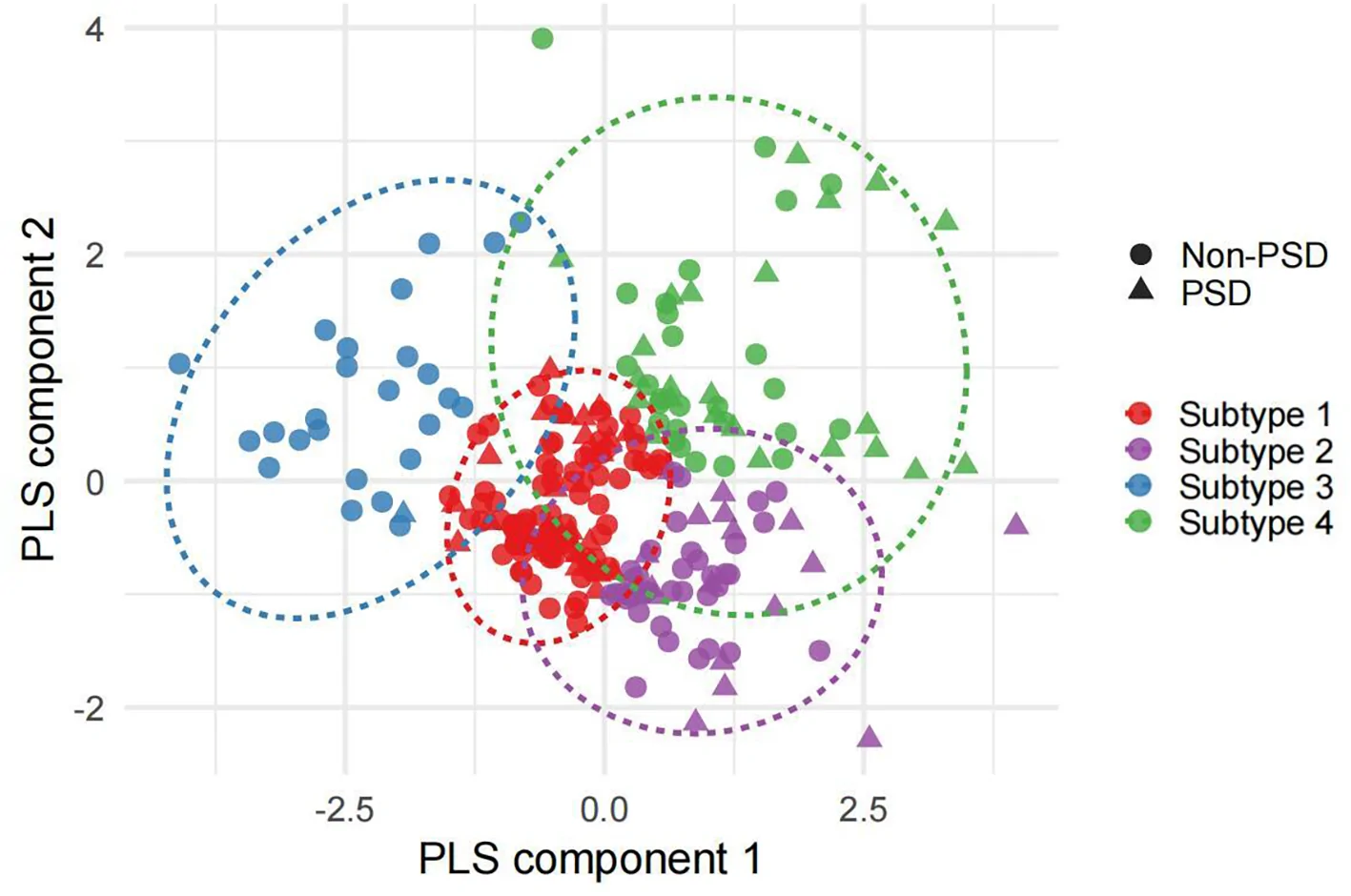
Distribution of functional–metabolic subtypes in the PLS-derived component space. Subtypes were derived using K-means clustering based on two principal components from PLS analysis. PLS component 1 reflects metabolic traits, while component 2 captures functional status. Colors represent different subtypes: subtype1 (red), physiological protection; subtype2 (purple), subclinical burden; subtype3 (blue), metabolically susceptible; subtype4 (green), dual-impaired. Dots indicate non-PSD patients, and triangles indicate PSD patients.

**Table 3:** Functional and metabolic characteristics of the four identified subtypes.

Subtype	Barthel	BMI	AIP	TyG	PSD rate
Subtype1 (n=26)	88.46	25.55	-0.06	2.00	3.85%
Subtype2 (n=116)	84.74	24.93	0.15	3.33	20.69%
Subtype3 (n=53)	82.26	24.33	0.38	4.13	32.08%
Subtype4 (n=50)	55.80	24.22	0.22	3.62	46%

AIP, atherogenic index of plasma; TyG, triglyceride-glucose.

**Table 4:** Multivariate logistic regression analysis of functional–metabolic subtypes and PSD.

Variables	β	S.E	Z	*P*	OR [95%CI]
Age	0.00	0.01	0.23	0.815	1.00 [0.98 ~ 1.03]
Male	-0.01	0.34	-0.03	0.980	0.99 [0.51 ~ 1.93]
NIHSS	0.07	0.05	1.46	0.144	1.07 [0.98 ~ 1.18]
Stroke history	0.55	0.33	1.68	0.094	1.74 [0.91 ~ 3.31]
Subtype(1 + 2)					
3	0.76	0.38	2.01	**0.044**	2.13 [1.02 ~ 4.46]
4	0.97	0.42	2.32	**0.020**	2.63 [1.16 ~ 5.96]

Values in bold indicate p<0.05. OR: Odds Ratio, CI: Confidence Interval; AIP, atherogenic index of plasma; TyG, triglyceride-glucose. subtype1, physiological protection; subtype2, subclinical burden, subtype3, metabolic susceptibility; subtype4, dual-impaired.

## 4 Discussion

PSD is one of the most common neuropsychiatric complications after stroke, significantly hindering patients’ rehabilitation, reducing their quality of life, and worsening long-term outcomes. Despite its clinical importance, the potential mechanisms underlying PSD remain incompletely understood. Existing studies have identified both functional impairment and metabolic dysregulation as independent risk factors for PSD. However, the combined effects of these two domains—and the heterogeneity in functional and metabolic phenotypes among stroke survivors—have not been systematically explored. This study aims to comprehensively characterize the integrated functional–metabolic profiles of post-stroke patients and examine their associations with the occurrence of PSD, thereby providing a theoretical basis for the early identification and stratification of high-risk individuals.

In our cohort, patients with PSD showed markedly lower Barthel index scores and higher values for several metabolic markers, indicating co-existing functional impairment and metabolic dysregulation. Further multivariate logistic regression analysis identified the Barthel Index (OR = 0.97, 95% CI: 0.95–0.99, *P* = 0.039) and the TyG index (OR = 2.04, 95% CI: 1.20–3.47, *P* = 0.009) as independent risk factors for PSD, further supporting the distinct associations of functional impairment and metabolic dysregulation with PSD. The Barthel Index reflects post-stroke ADL; a decline in this score not only indicates reduced self-care ability but may also reflect increased psychological burden and reduced confidence in recovery (22). Insulin resistance, indexed by TyG, is tightly linked to inflammation and oxidative stress and may provoke depressive symptoms by disrupting neurotransmitter metabolism and neuroplasticity (2). By contrast, the AIP was not a significant predictor in our model—likely because its relationship with PSD is nonlinear. AIP may trigger inflammatory or endocrine cascades only beyond a threshold, a pattern that linear models can undershoot. In addition, AIP is a derived index calculated from TG and HDL rather than a directly measured biomarker; therefore, its value is influenced not only by the two underlying laboratory measurements but may also inherit and potentially amplify their analytical errors. Holmes et al. demonstrated that, under the conditions of their illustrative example, analytical errors in TG and HDL-C measurements could result in an approximately ±0.04 range of variation in AIP at the 95% level (23). Such error propagation may increase the measurement uncertainty of AIP and, to some extent, attenuate the observable nonlinear association between AIP and PSD. Notably, findings on AIP remain inconsistent: a community-based study of older adults in China reported no significant nonlinearity between AIP and PSD (24). Such discrepancies could stem from differences in sample characteristics or covariate adjustment strategies, suggesting that contextual factors modulate the AIP-PSD association. Larger, multi-center studies are needed to clarify these mechanisms.

These findings underscore the importance of simultaneously assessing functional status and metabolic phenotype in the early stages following stroke. Although previous studies have highlighted associations between functional impairment and metabolic dysregulation in stroke patients, limited evidence exists regarding their combined influence on the risk of PSD. Most existing literature focuses either on the impact of metabolic abnormalities on cardiovascular or cerebrovascular outcomes, or considers functional impairment as an isolated risk factor for PSD, without systematically addressing the interaction between these two domains. To bridge this gap, the present study is the first to apply PLS-K-means clustering to categorize stroke patients based on integrated functional and metabolic indicators, including the Barthel index, BMI, AIP, and TyG index. Four distinct functional–metabolic subtypes were identified: physiological protection type, subclinical burden type, metabolic susceptibility type, and dual-impaired type, revealing substantial heterogeneity across patients. After adjusting for age, sex, and other covariates, subtype 1 and 2 (characterized by preserved function and low metabolic burden) were combined as the reference group to evaluate the independent impact of functional–metabolic subtypes on the risk of PSD. Both metabolically susceptible subtype (OR = 2.13, 95% CI: 1.02–4.46, *P* = 0.044) and dual-impaired subtype (OR = 2.63, 95% CI: 1.16–5.96, p = 0.020) showed significantly increased risks of PSD. These findings confirm that functional–metabolic phenotypes independently contribute to PSD risk stratification, suggesting that coexisting functional impairment and metabolic dysregulation jointly shape post-stroke psychological vulnerability.

Specifically, the metabolically susceptible subtype demonstrated unique risk features. Despite exhibiting relatively preserved functional status, this subtype showed a markedly elevated metabolic burden—characterized by higher TyG and AIP levels—alongside a PSD incidence of 32.08%. These findings suggest that even in the absence of overt functional impairments, underlying inflammatory and oxidative stress may increase vulnerability to post-stroke depression. As surrogate markers of insulin resistance and lipid metabolism, respectively, the TyG index and AIP reflect metabolic dysregulation linked to chronic inflammation (25, 26). Moreover, studies have reported strong associations between TyG and increased white blood cell counts or hsCRP levels, suggesting a potential inflammatory pathway linking TyG to PSD risk (27). Similarly, in patients with myocardial infarction, higher TyG levels have been associated with elevated hsCAR, and the combined use of TyG and the high-sensitivity C-reactive protein–albumin ratio enhanced cardiovascular risk prediction (28). Elevated AIP, reflecting an imbalanced TG/HDL-C ratio, has also been recognized as a marker of low-grade systemic inflammation. A recent population-based study reported a significant positive association between AIP and the systemic immune-inflammation index (β = 32.497, 95% CI: 5.425–59.569, *P* = 0.021), further supporting its role in identifying subclinical inflammatory states (29).

Inflammatory mechanisms are central to ischemic brain injury, and an imbalance between pro- and anti-inflammatory mediators can worsen neurological outcomes. Such dysregulation has been implicated in both PSD and primary major depressive disorder, suggesting potentially shared biological pathways (30, 31). Although overt clinical signs of systemic inflammation are often absent, persistent activation of central inflammatory pathways can induce neuro-immune dysfunction and heighten emotional vulnerability. Recent animal studies further support this pathological pathway. Following stroke, alleviating hippocampal inflammation promotes neuronal repair and significantly improves depressive-like symptoms in post-stroke mouse models (32). Metabolic dysregulation further amplifies this cascade by provoking oxidative stress, compromising blood–brain-barrier integrity, and exacerbating neuro-inflammation and functional deficits (33). Consistent with this, Wen reported that early-phase oxidative stress—reflected by reduced superoxide-dismutase activity and elevated malondialdehyde levels on day 3 after stroke—accurately predicted PSD within two weeks (17). Collectively, these observations describe a “metabolic–inflammation–neural” axis that explains why patients in the metabolically susceptible subtype can appear functionally intact yet remain intrinsically vulnerable to depression. Recognizing this high-risk but clinically covert phenotype underscores the need for early screening and targeted intervention, emphasizing that PSD is driven not only by residual disability but also by inflammation- and neurotransmitter-related sequelae of metabolic dysregulation.

Additionally, the dual-impaired subtype was characterized by severe dysfunction and pronounced metabolic abnormalities, exhibiting the highest incidence of PSD at 46%. Patients in this group may be particularly susceptible to psychological distress due to the combined effects of significant functional impairment—which foster dependency and social isolation—and metabolic dysregulation such as insulin resistance and dyslipidemia. These coexisting conditions can jointly disrupt emotional regulation, resulting in heightened psychophysiological vulnerability. ADL serve as a critical indicator of stroke recovery. Impairments in ADL not only reflect substantial neurological damage but also indicate the need for considerable assistance with basic life tasks such as eating, toileting, dressing, and walking. This level of dependence often leads to negative emotional states, including feelings of helplessness, reduced self-esteem, and social withdrawal (9). Previous research has demonstrated a significant association between lower ADL scores and an increased risk of PSD, suggesting that ADL is a reliable predictor of PSD during both the acute and rehabilitation phases (34). Moreover, ADL dependency may hinder participation in rehabilitation and limit social engagement, thereby exacerbating depressive symptoms and promoting maladaptive cognitive responses (8). In addition, metabolic abnormalities have been closely linked to functional decline. Liaw reported a strong correlation between metabolic dysregulation and ADL dependency, showing that the risk of disability across multiple functional domains increases significantly with the number of metabolic syndrome components (35). Functional impairment is often accompanied by chronic inflammation and physical decline, which together can create a self-reinforcing cycle among metabolic dysregulation, inflammation, and reduced function. It is also important to emphasize that these associations may be bidirectional, as depressive symptoms after stroke could, in turn, adversely affect metabolic status and functional recovery (36). This bidirectional relationship may partly explain the close coexistence of functional impairment, metabolic burden, and depressive symptoms observed in these patients. Therefore, for stroke patients with functional impairment, greater attention should be paid to the combined effects of functional deficits and metabolic abnormalities on mental health. Early psychological risk assessment and targeted interventions should be integral components of comprehensive post-stroke care.

For the first time, this study identified distinct functional–metabolic subtypes among stroke patients by integrating functional and metabolic dimensions. Notably, individuals classified as metabolically susceptible or dual-impaired were found to have a significantly higher risk of developing PSD. These findings not only enhance our understanding of the potential mechanistic hypotheses associated with PSD but also provide novel insights into the early identification and stratified management of high-risk populations. In clinical practice, incorporating both functional status and metabolic burden as comprehensive assessment indicators may support the development of more targeted psychological interventions tailored to specific patient phenotypes, thereby reducing PSD incidence and improving long-term outcomes.

However, several limitations should be acknowledged. First, the cross-sectional nature of the study precludes inference of a causal relationship between metabolic dysregulation and PSD development. Second, the relatively limited sample size, particularly within the AIP quartile groups, together with potential residual confounding and measurement noise, may have further reduced the statistical power, warranting validation in larger prospective cohorts. Third, PSD was assessed only once within the first month after stroke, which limited our ability to capture temporal changes and chronicity of depressive symptoms or to distinguish between early- and late-onset PSD. In addition, although patients with a documented history of depression or other major psychiatric disorders were excluded, pre-stroke subthreshold depressive symptoms could not be completely ruled out. Finally, some potentially relevant clinical variables, including comorbidities, pre-stroke psychiatric history, and medication use, were not comprehensively captured, which limited our ability to adequately adjust for potential confounding factors.

## 5 Conclusion

This study identified reduced functional independence and elevated TyG index levels as independent predictors of PSD, underscoring the combined effect of functional impairment and metabolic dysregulation on psychological vulnerability after stroke. In addition, by applying a PLS-K-means clustering approach based on the Barthel Index, BMI, AIP, and TyG index, we revealed significant heterogeneity in the functional–metabolic profiles of stroke survivors. Individuals classified as metabolically susceptible or dual-impaired exhibited a markedly higher risk of PSD. These findings highlight the importance of incorporating both functional and metabolic assessments into early screening and personalized management strategies for PSD. Future interventions should be tailored to specific phenotypes in order to optimize psychological outcomes and rehabilitation trajectories in stroke survivors.

## Data Availability

The original contributions presented in the study are included in the article/**Supplementary Material**. Further inquiries can be directed to the corresponding authors.
