# Supplementary material for: Combined effect of functional impairment and metabolic dysregulation on post-stroke depression risk
Source: Front Psychiatry. 2026 Oct 16;17:1843802. doi: 10.3389/fpsyt.2026.1843802 (PMC13720283; doi:10.3389/fpsyt.2026.1843802)
Supplement: Supplementary file 1 [file SupplementaryFile1.docx]

**Table S1** Loading Matrix and Variance Summary

|  | Component 1 | Component 2 |
| --- | --- | --- |
| Barthel | -0.52 | -0.791 |
| BMI | -0.124 | -0.066 |
| TyG | 0.657 | -0.463 |
| AIP | 0.552 | -0.476 |
|  |  |  |
| SS loadings | 1.022 | 1.07 |
| Proportion Var | 0.255 | 0.268 |
| Cumulative Var | 0.255 | 0.523 |

Notes: AIP, atherogenic index of plasma; TyG, triglyceride-glucose.

**Supplementary Fig S1.** Determination of the optimal number of clusters.

Notes: A: Elbow plot based on the within-cluster sum of squares (WSS), showing diminishing reductions in WSS around K = 4. B: D-index values and the corresponding second-order differences across candidate cluster numbers; the second-order difference reached its maximum at K = 4. C: Number of NbClust indices supporting each candidate number of clusters. K = 4 received the greatest support (9 indices), supporting the four-cluster solution.
